# Supplementary material for: Albizia julibrissin Exerts Anti-Obesity Effects by Inducing the Browning of 3T3L1 White Adipocytes
Source: Int J Mol Sci. 2023 Jul 15;24(14):11496. doi: 10.3390/ijms241411496 (PMC10380714; doi:10.3390/ijms241411496)
Supplement: Supplementary file 1 [file ijms-24-11496-s001.zip › ijms-2475563-supplementary.pdf]

*Article*

# *Albizia julibrissin* Exerts Anti-Obesity Effects by Inducing the Browning of 3T3L1 White Adipocytes

**Yuna Kim [1], Hyanggi Ji [1], Dehun Ryu [1], Eunae Cho [1], Deokhoon Park [1] and Eunsun Jung [1,*]**

[1] Biospectrum Life Science Institute, Yongin 16827, Korea; bioyn@biospectrum.com (Y.K.); biocr@biospectrum.com (H.J.); biozr@biospectrum.com (E.C.); biosc@biospectrum.com (D.R.); pdh@biospectrum.com (D.P.)

* Correspondence: bioso@biospectrum.com (E.J); Tel.: +82-70-5117-0043 (E.J.)

## Supplementary Materials

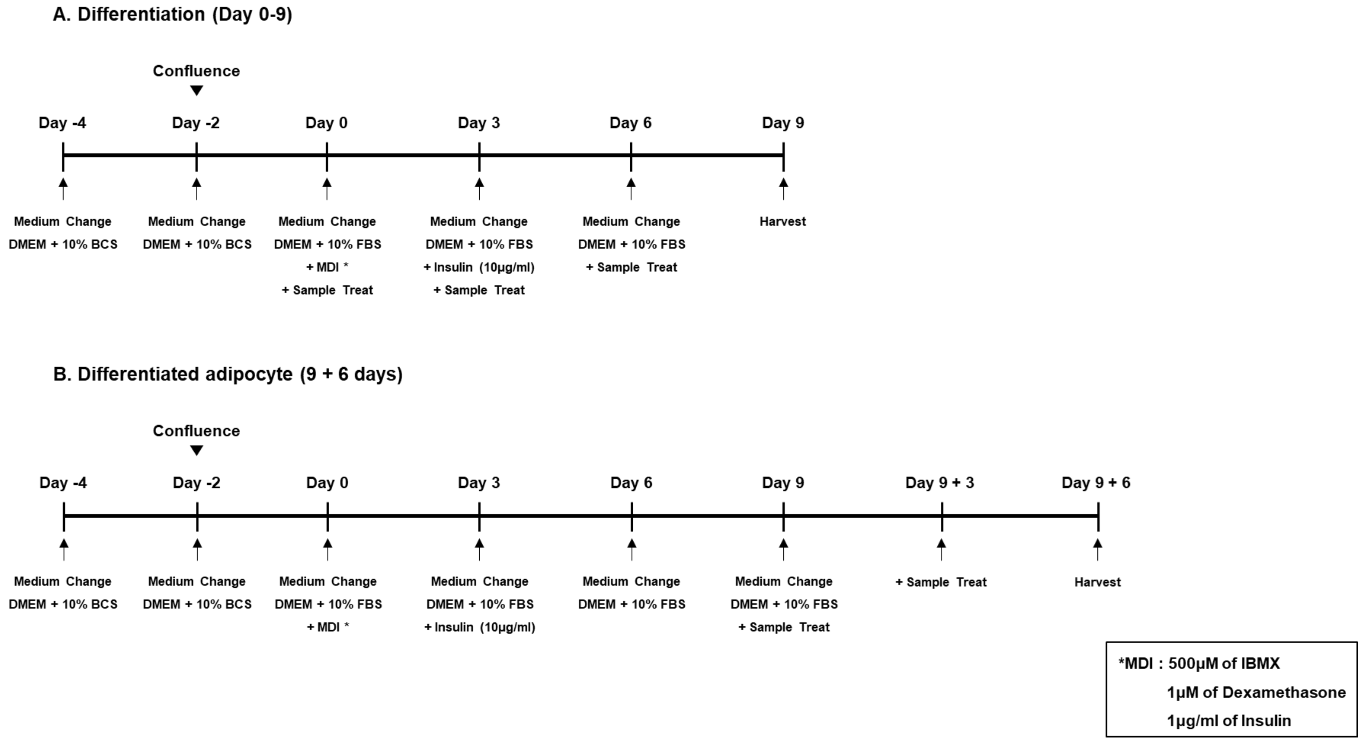


**Figure S1.** Procedure of adipocyte differentiation. (A) Differentiation with AJLE from pre-adipocytes to mature adipocyte. (B) Treat with AJLE after differentiated.

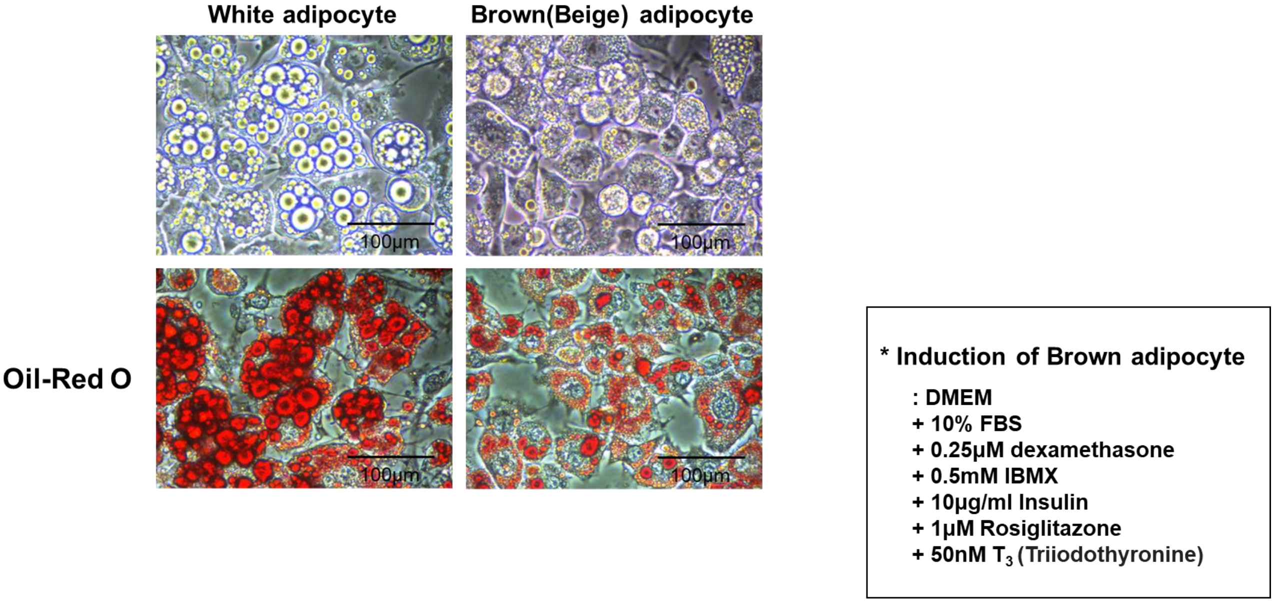


**Figure S2.** Differentiation to brown adipocytes. Brown adipocyte differentiation was induced by treating with 1 μM of rosiglitazone and 50 nM of $T_3$. * Morphological confirmation revealed the induction of brown adipocyte formation compared to differentiated white adipocytes.

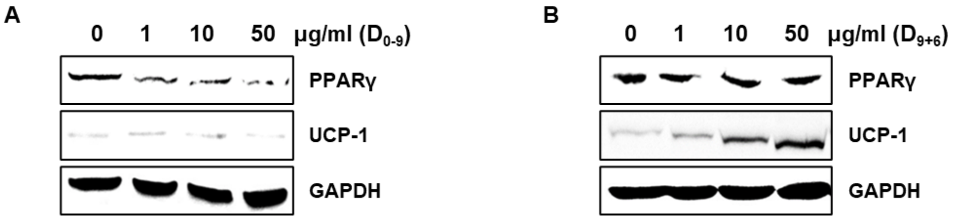


**Figure S3.** AJLE not differentiates to brown adipocytes but converses white adipocytes to brown adipocytes. (A) During the differentiation induction stage, it was confirmed that treatment with AJLE did not induce differentiation into brown adipocytes, as evidenced by the expression of PPARγ and UCP-1. (B) After inducing differentiation into white adipocytes, treatment with AJLE revealed the expression of brown adipocyte-associated proteins, confirming the efficacy of AJLE in converting white into brown adipocyte.

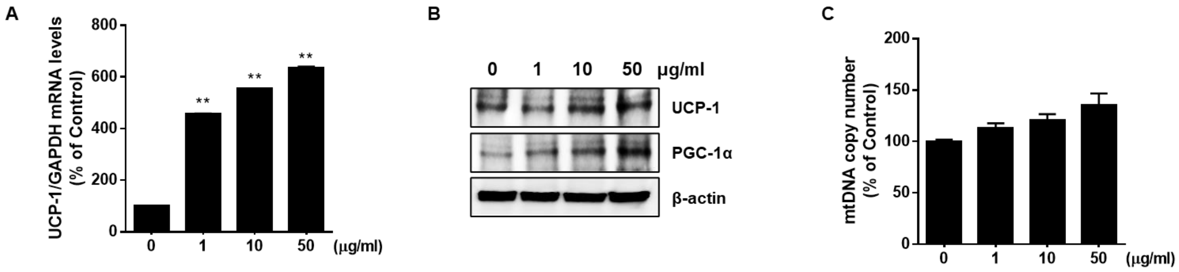


**Figure S4.** AJLE regulates mitochondrial metabolism in C2C12 skeletal muscle cells. (A) Treatment with AJLE in skeletal muscle cells resulted in an increase in UCP-1 mRNA expression. (B) The protein expression of UCP-1 and PGC-1$\alpha$ was confirmed to be increased following AJLE treatment. (C) It was observed that mitochondrial copy number increased in response to AJLE treatment. The results are expressed as the mean ± standard deviation (SD) (n = 3). * $p < 0.05$; ** $p < 0.01$, compared to control.
